# Parent and child experiences of research participation and retention intentions in a Japanese birth cohort: insights from the Japan Environment and Children’s Study (JECS)

**DOI:** 10.1265/ehpm.25-00355

**Published:** 2026-07-25

**Authors:** Tomoko Okabe, Yoshimitsu Takahashi, Mayumi Toyama, Yoshitaka Nishikawa, Kyoko Hirabayashi, Kumiko Kanatani, Fusako Niwa, Takeo Nakayama

**Affiliations:** 1 Department of Health Informatics, Kyoto University School of Public Health, Kyoto, Japan; 2 Department of Implementation Science in Public Health, Kyoto University School of Public Health, Kyoto, Japan; 3 Japan Environment and Children’s Study Kyoto Regional Center, Kyoto University Graduate School of Medicine, Kyoto, Japan

**Keywords:** Birth cohort, Participant experience, Research engagement, Retention intention, Patient and public involvement, Children’s autonomy

## Abstract

**Background:**

Long-term birth cohort studies depend on sustained participant engagement. In such studies, children are not only observed over time but also increasingly become participants who experience, interpret, and evaluate their own involvement in research. However, research on continued participation has focused mainly on parental perspectives, with limited attention to children’s own views or to similarities and differences between parents and children. Against the growing recognition that people in medical research should be understood as participants rather than passive subjects, this study examined research participation experiences and retention intentions in both parents and children within a Japanese birth cohort.

**Methods:**

We conducted a questionnaire survey from September to October 2024 within the Japan Environment and Children’s Study (JECS). The primary outcome was retention intention. Factors associated with retention intention were analyzed separately for parents and children using ordinal logistic regression. Parent-child concordance in retention intention was assessed descriptively using weighted Cohen’s kappa.

**Results:**

Among 1,008 eligible parents of fifth-grade children, 485 responded (response rate, 44%). A large majority of parents (92%) and children (79%) reported willingness to continue participation. Parent–child responses showed moderate agreement (κ = 0.46). In separate adjusted ordinal logistic regression models, stronger parental retention intention was associated with lower perceived questionnaire burden (adjusted odds ratio [aOR]: 5.58, 95% confidence interval [CI]: 3.40–9.17), fewer concerns about possible disagreement with their child (aOR: 5.75, 95% CI: 3.51–9.42), and less difficulty scheduling study activities (aOR: 5.80, 95% CI: 3.50–9.63). In children, stronger retention intention was associated with anticipated gifts (aOR: 7.38, 95% CI: 2.93–18.56), talking with friends about the study (aOR: 6.02, 95% CI: 2.25–16.09), and a desire for peer interaction (aOR: 6.72, 95% CI: 3.39–13.29).

**Conclusions:**

Parents and children both showed generally positive retention intentions, but disagreement was not negligible. Their retention intentions were associated with different participation-related experiences, suggesting that long-term birth cohort studies should attend to participants’ perspectives in age-specific ways. Understanding research participation through both parental and child experiences may help inform more participant-centered engagement strategies and support the quality of long-term cohort research.

**Supplementary information:**

The online version contains supplementary material available at https://doi.org/10.1265/ehpm.25-00355.

## Graphical Abstract

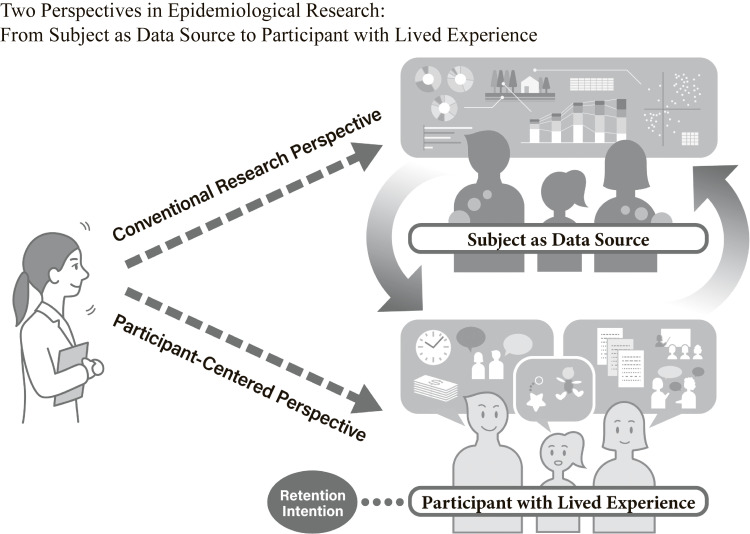

## Introduction

Medical research has long aimed to generate valid and reproducible answers to researchers’ questions. In that process, people involved in research have often been treated primarily as objects of observation for hypothesis testing. However, the 2024 revision of the World Medical Association Declaration of Helsinki replaced the term “human subjects” with “human participants.” The revision workgroup explained this change as reflecting respect for the rights, agency, and importance of individuals involved in research, and the revised Declaration further calls for meaningful engagement with potential and enrolled participants and their communities before, during, and after research [1, 2]. This revision suggests that people in research should be understood not merely as persons to be protected, but as individuals whose perspectives matter to the conduct of research itself.

This shift has implications not only for research ethics but also for the questions researchers ask. Researchers may ask not only how to test their hypotheses, but also how people experience research participation, what meanings they attach to it, and what attitudes they form toward research and researchers. This question is scientifically important as well as ethically important, because participants’ experiences and trust may influence willingness to remain in a study and cooperate actively, thereby affecting follow-up, response rates, item completion, and participation in additional procedures. In longitudinal cohort studies, engagement is shaped not only by one-way information provision but also by reciprocal relationships, trust, and sustained interaction over time [3].

These issues are particularly important in birth cohort research. Birth cohort studies follow individuals from the prenatal or birth period over long periods to identify determinants of health and development, and their scientific value depends heavily on successful long-term follow-up [4, 5]. Continued engagement is therefore not only an operational challenge but a condition for the success of the study design itself. Birth cohort studies also differ from adult cohort studies in another key respect: children enter research through parental permission or proxy consent rather than their own direct consent. As they grow, however, they become increasingly aware that they are participating in research, begin to interpret that experience in their own ways, and may gradually form their own views about continued participation. Prior work suggests that engagement in longitudinal studies may shift from parental decision-making toward more autonomous judgment by participants themselves [3, 6].

Previous studies have identified several factors associated with continued participation in birth cohort studies, including understanding study aims, perceived family benefit, reduced burden, trust in researchers, and the quality of communication [7–10]. However, most of this evidence is based primarily on parents’ responses. Much less is known about how children themselves experience participation, what meanings they assign to it, and where their intentions to continue participation converge with or diverge from those of their parents [11, 12]. If people in research are understood as “participants” rather than merely “subjects,” examining the experiences and retention intentions of both parents and children is not simply a matter of improving retention. Rather, it is a scientific question about the formation of participation-related agency and the evolving relationship between people and research over time [1–3, 6].

Against this background, the present study used the Japan Environment and Children’s Study (JECS), one of the major nationwide birth cohort studies in Japan, as a concrete case to examine parents’ and children’s experiences of research participation and their intentions regarding continued participation. By clarifying where parents’ and children’s intentions converge and where they diverge, this study aims to provide a participant-centered perspective on birth cohort research and to contribute to understanding how long-term research quality may be supported through attention to participants’ lived experiences of research participation [13].

## Methods

### Study design

This cross-sectional study used an online questionnaire. This report adheres to the Checklist for Reporting Of Survey Studies (CROSS) guidelines [14] and the Strengthening the Reporting of Observational Studies in Epidemiology (STROBE) statement [15].

### Study cohort

This study involved participants in the Japan Environment and Children’s Study (JECS). The JECS is a nationwide birth cohort study launched in 2011 to examine environmental influences on child health and development. About 100,000 mother–child pairs were enrolled with data collected from the prenatal period onward.

The JECS is led by the National Institute for Environmental Studies in collaboration with 15 regional centers, mainly universities and research institutes. Study details have been described elsewhere by Kawamoto et al. (2014) [16]. This study was conducted as Adjunct Study of the JECS by the Ministry of the Environment, Japan.

### Study settings and participants

In JECS, participants regularly complete postal questionnaires and undergo standardized “school-age examinations” in the second and sixth grades, which include physical and neurodevelopmental assessments. Additionally, approximately 5% of participants receive detailed assessments via home visits, including residential environment evaluations and medical examinations [16].

This study targeted parent–child pairs followed by Kyoto Regional Center (Kyoto and Nagahama areas) and whose children entered fifth grade (ages 10–11) in 2024. The eligible sample included 693 pairs from Kyoto and 415 from Nagahama. Originally, JECS follow-up was planned until age 13, but at the time of study, plans were underway to extend it to age 18. Although formal re-consent had not yet been implemented, families were expected to make such a decision in the near future. Fifth grade marks a development stage where children begin forming independent views and decision-making abilities, making it a key point for examining their research engagement [17–20].

### Survey administration

Between September 3 and October 20, 2024, parents with internet access were invited by mail to complete an online questionnaire about their experiences and retention intention. Informed consent was obtained electronically via the Kyoto Regional Center website, where participants accessed the study description and submitted responses using a unique ID. A reminder postcard was sent in late September.

### Study preparation

We developed the questionnaire based on interviews with four parent–child pairs (one grade above the target group) and consultations with experienced JECS staff to ensure clarity and minimize burden.

### Data collection methods

We administered a three-part online questionnaire to parent–child pairs. (“P” and “C” indicate parent and child items, respectively.) In Section 1, parents answered 16 items on participation experience (P3-4: 6-point scale with “not applicable”; others 5-point scale), and one item on retention intention (P16, 5-point scale). In Section 2, children responded to 14 items about their experiences (C1-C3: 6-point scale; others used 5-point scale) and one item on retention intention (C17, 5-point scale). All items were treated as ordinal variables. Both sections included open-ended questions on positive (P14, C15), and negative experiences (P15, C16), and concerns about continued participation (P20, C18). Section 3 asked parents to rate their likelihood of recommending the study (0–10 scale) [21] and provide demographic data. Open-ended questions and recommendation item were included for exploratory purposes but not analyzed in this study.

The survey was completed using smartphones or tablets. After parental consent, parents answered Section 1, children completed Section 2 without seeing parental responses in the standard survey flow, and parents then answered Section 3. Although backward navigation was technically possible, prior responses could only be viewed if participants intentionally returned to earlier screens, and participants were instructed to proceed sequentially to ensure independent responses.

### Statistical analysis

We first summarized the distribution of 16 parent items and 14 child experience items, along with 5-point retention intention scores (excluding “not applicable” responses). Retention intention was defined as the main outcome. Parent–child agreement was also examined using quadratic weighted Cohen’s kappa, exact agreement and larger parent-child discrepancies (differing by two to four categories) according to 5-point retention scores. After collapsing 5-point retention intention score into these categories (intend, neutral, do not intend), ordinal logistic regression was conducted. For parents, we fitted a series of separate models, each including one parent experience item as the predictor of interest; children’s retention intention was additionally included as a parallel reference variable for comparison. For children, we similarly fitted separate models for each child experience item, with parental retention intention also examined as a parallel reference variable. Experience items were modeled using three categories (Negative/Neutral/Positive), retaining neutral responses as a separate category. Negative was used as the reference category. Multivariable models adjusted for parent’s age, child’s sex, household income (four levels), and parent’s education (three levels). Thus, each parental model included one predictor of interest plus four covariates, rather than all 16 experience items simultaneously. Parent sex was not included as a covariate because respondents were predominantly female. Adjustment was limited to small number of key demographics variables to reduce the risk of overfitting and maintain interpretability. Correlations among the four covariates were examined, and no strong correlations were observed. The proportional-odds assumption was tested with the Brant test, and model fit assessed by likelihood-ratio tests. Complete-case analysis was used. Responses such as “did not participate”, “don’t know”, “don’t recall,” were treated as missing, except for “don’t know” on detailed assessment item, which was coded as “no”.

Analyses were conducted using JMP Pro 18 (SAS Institute Inc., Cary, NC, USA) and R (Version 4.4.0; R Core Team, Vienna, Austria).

## Results

### Respondent characteristics

**Fig. 1:**
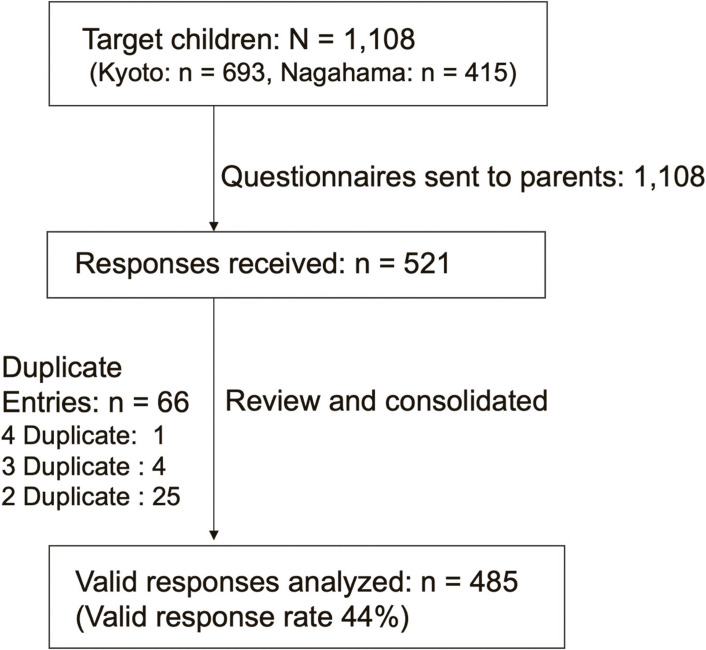
Flow of questionnaire distribution, response received, and final valid responses Flow diagram showing the distribution process of the parent and child questionnaires in the JECS cohort, the number of responses received, and the final number of valid responses included in the analysis. Includes two responses from one parent of twins.

Of 1108 questionnaires mailed, 522 were returned. After excluding 66 duplicate submissions (retaining the latest or more complete version) and one blank response, 485 were analyzed (valid response rate: 44%) (Fig. 1). Twin cases were counted as separate pairs. Among respondents (n = 485), 98% (n = 475) were mothers. Regarding age, 71% were in their 40s and 18% in their 30s. Children were nearly evenly distributed by sex (49% boys, 51% girls). 66 children (14%) had participated in the JECS detailed assessment. School-age health examination participation was reported by 79% (n = 383), and 75% (n = 364) of parents reported completing the annual JECS questionnaires “every year without fail.” Travel time to examination site was <30 minutes for 56%, and ≥2 hours for 4%. Regarding socioeconomic status, 39% (n = 191) reported annual household incomes of 6–10 million yen, and 42% (n = 203) of parents had a university degree (Table 1).

**Table 1:** Demographic characteristics of parent–child participants (N = 485)

**Characteristics**	**Variables**	**n**	**(%)**
Sex (parent)	Male	9	(2)
	Female	475	(98)
	Missing	1	(0)

Sex (children)	Male	238	(49)
	Female	246	(51)
	Missing	1	(0)

Age group^1)^ (Parent)	30–39	89	(18)
	40–49	345	(71)
	50–59	49	(10)
	60 and over	1	(0)
	Missing	1	(0)

Participation in detailed assessments^2)^	Yes	66	(14)
	No	356	(73)
	Don’t know	62	(13)
	Missing	1	(0)

Participation in school-age examination^3)^	Yes	383	(79)
	No	90	(18)
	Don’t know	12	(2)

Response to regular questionnaires	Responded every year	364	(75)
	Responded almost every year	113	(23)
	Rarely Responded	5	(1)
	Never Responded	1	(0)
	Missing	2	(0)

Children’s extracurricular activities (days per week)	7	15	(3)
	6	30	(6)
	5	58	(12)
	4	76	(16)
	3	81	(17)
	2	85	(18)
	1	77	(16)
	0	62	(13)
	Missing	1	(0)

Duration to reach examination venue	≦30 minutes	272	(56)
	>30 minutes to 1 hour	132	(27)
	>1 hour to 2 hours	21	(4)
	>2 hours	19	(4)
	Don’t know	37	(8)
	Missing	4	(1)

Household income (million JPY)	≦2	7	(1)
	2–4	29	(6)
	4–6	80	(17)
	6–8	93	(19)
	8–10	98	(20)
	10–12	48	(10)
	12–14	26	(5)
	15–20	12	(3)
	>20	15	(3)
	Prefer not to answer	67	(14)
	Missing	10	(2)

Parents’ educational background	Graduate School	35	(7)
	University	203	(42)
	Junior College	102	(21)
	Vocational School	74	(15)
	High School	47	(10)
	Technical College	8	(2)
	Junior High School	8	(2)
	Prefer not to answer	7	(2)
	Missing	1	(0)

**1)** Children were in the fifth grade of elementary school in Japan as of 2024, typically aged 10–11 years.

**2)** Approximately 5% of all JECS participants (about 5,000 children and their caregivers) are selected for detailed assessments, which include home visits to evaluate residential and living environments, neurodevelopmental testing, and medical examinations of health and growth [20].

**3)** The school-age survey is conducted to assess the health and developmental status of children in the second and sixth grades of elementary school. Participants are invited to designated local venues, where standardized equipment and procedures are used nationwide for physical measurements and neurodevelopmental assessments [20].

### Retention intention and parent–child agreement

Retention intention was reported by 92% of parents (443/483) and 79% of children (384/484) (Table 2-1). Across the original five-point scale, parent–child responses showed moderate [22] agreement overall (quadratically weighted κ = 0.46). For descriptive context, exact agreement on the original five-point scale was 53% (256 pairs) (Table 2-2), whereas responses differing by 2–4 categories accounted for 10% (50 pairs).

**Table 2-1:** Concordance of retention intentions between parents and children (3 categories, N = 482)

		**Parent n (%)**			
		**Do not intend**	**Neutral**	**Intend**	**Total**
**Children** **n (%)**	**Do not intend**	11 (3)	2 (0)	14 (3)	27 (6)
	**Neutral**	5 (1)	7 (1)	60 (12)	72 (15)
	**Intend**	3 (1)	11 (2)	369 (77)	383 (79)
	**Total**	19 (4)	20 (4)	443 (92)	482 (100)

Intend: Definitely intend to continue & somewhat intend to continue

Do not intend: Somewhat do not intend and definitely do not intend

**Table 2-2:** Concordance of retention intentions between parents and children (N = 482)

		**Parents n (%)**					
		**1**	**2**	**3**	**4**	**5**	**Total**
**Children** **n (%)**	**1**	3 (1)	5 (1)	0 (0)	3 (1)	2 (0)	13 (3)
	**2**	1 (0)	2 (0)	2 (0)	6 (1)	3 (1)	14 (3)
	**3**	0 (0)	5 (1)	7 (2)	30 (6)	30 (6)	72 (15)
	**4**	0 (0)	1 (0)	8 (2)	48 (10)	81 (17)	138 (29)
	**5**	0 (0)	2 (0)	3 (1)	44 (9)	196 (41)	245 (51)
	**Total**	4 (1)	15 (3)	20 (4)	131 (27)	312 (65)	482 (100)

1. Definitely do not intend

2. Somewhat do not intend

3. Neutral

4. Somewhat intend to continue

5. Definitely intend to continue

*After excluding two parent and one child missing responses, 482 complete dyads were analyzed.

*The quadratically weighted kappa coefficient was 0.46.

### Participation experiences and parent–child agreement

Parents most frequently reported positive experiences with accompanying their child to the school-age examination (89%) and with staff support during the visit (98%). High approval was also observed for information-related items including interest in study results (90%), and clarity of explanations (81%) (Table 3). In contrast, only 53% looked forward to JECS newsletter. While 82% had discussed the study with their child, 77% reported not having talked with other participating parents.

**Table 3:** Parental participation experiences (N = 485)

**No.**	**Item (Summary)**	**Experience**			
		**Positive**	**Neutral**	**Negative**	**Missing**
		**n (%)**	**n (%)**	**n (%)**	**n (%)**
P1	Was responding to the regular questionnaires burdensome?	319 (65.8)	74 (15.3)	92 (19.0)	0 (0)
P2	Was scheduling the school-age examination burdensome?	308 (63.9)	102 (21.2)	72 (14.9)	3 (1)
P3^1)^	Did you enjoy coming to the venue with your child for the school-age examination? *	349 (89.0)	39 (10.0)	4 (1.0)	1 (0)
P4^1)^	Was the staff’s response during the school-age examination courteous? **	385 (98.5)	6 (1.5)	0 (0.0)	2 (1)
P5	How was the JECS newsletter?	256 (52.8)	177 (36.5)	52 (10.7)	0 (0)
P6^2)^	Have you seen JECS mentioned in mass media (TV, newspapers)? ***	43 (30.5)	89 (63.1)	9 (6.4)	2 (1)
P7	How interested are you in the returned results from JECS?	435 (89.9)	33 (6.8)	16 (3.3)	1 (0)
P8	Was the explanation accompanying the returned results easy to understand?	392 (81.0)	84 (17.4)	8 (1.7)	1 (0)
P9	Have you talked with your child about the JECS Study?	398 (82.1)	21 (4.3)	66 (13.6)	0 (0)
P10	Do you feel your child has become more interested in health or society through JECS?	142 (29.4)	176 (36.4)	165 (34.2)	2 (0)
P11	Have you ever wanted to talk to someone about your participation in JECS?	256 (52.9)	111 (22.9)	117 (24.2)	1 (0)
P12	Have you talked with other parents participating in JECS?	46 (9.8)	62 (13.1)	364 (77.1)	0 (0)
P13	What do you think about increasing opportunities to talk with other parents?	159 (32.9)	281 (58.1)	44 (9.1)	1 (0)
P17	Do you think your child may come to disagree with your intention to continue participation?	227 (47.3)	113 (23.5)	140 (29.2)	5 (1)
P18	Do you anticipate difficulty continuing due to increased time demands in junior/ high school?	184 (38.3)	127 (26.5)	169 (35.2)	5 (1)
P19	Are you concerned about your relationship with your child during adolescence?	215 (44.8)	101 (21.0)	164 (34.2)	5 (1)

1) Participants who missed the school-age examination were excluded from these analyses (n = 92).

2) Participants who answered “Do not remember” were excluded from the analyses of P6 (n = 342) and P12 (n = 13).

Children also reported positive experiences; 95% were satisfied with staff support, 77% appreciated being accompanied, and 69% viewed examination positively. While 82% looked forward to the gift campaign, only 23% anticipated the newsletter and 36% were interested in events. About 63% had discussed JECS with their parent, but only 7% had talked to friends. 66% wanted to decide about participation themselves (Table 4).

**Table 4:** Children’s participation experiences (N = 485)

**No.**	**Item (Summary)**	**Experience**			
		**Positive**	**Neutral**	**Negative**	**Missing**
		**n (%)**	**n (%)**	**n (%)**	**n (%)**
C1^1)^	How was your experience receiving the examination at the venue in 2nd grade?	267 (68.5)	61 (15.6)	62 (15.9)	2 (1)
C2^1)^	Was the JECS staff courteous during the school-age examination?	372 (95.4)	17 (4.4)	1 (0.3)	2 (1)
C3^1)^	Did you enjoy going to the school-age examination with your parent or guardian?	300 (76.7)	76 (19.4)	15 (3.8)	3 (1)
C4	Do you want to go to the examination venue alone when you become a junior high student?	82 (17.0)	139 (28.8)	262 (54.2)	2 (0)
C5	Did you look forward to the gift campaign?	399 (82.4)	68 (14.1)	17 (3.5)	1 (0)
C6	Did you enjoy receiving the JECS newsletter?	113 (23.4)	250 (51.7)	121 (25.0)	1 (0)
C7	Would you like to participate in events hosted by JECS?	171 (35.6)	180 (37.5)	129 (26.9)	5 (1)
C8	Do you feel that participating in the study contributes to society?	256 (53.2)	148 (30.8)	77 (16.0)	4 (1)
C9	Have you talked with your parent(s) about the JECS Study?	307 (63.4)	37 (7.6)	140 (28.9)	1 (0)
C10	Has your interest in health, society, or the environment increased through the study?	192 (39.8)	160 (33.2)	130 (27.0)	3 (1)
C11	Have you talked about the JECS Study with your friends?	33 (6.8)	81 (16.7)	371 (76.5)	0 (0)
C12	Would you like to talk with children from other schools who are participating in the study?	72 (14.9)	128 (26.5)	284 (58.7)	1 (0)
C13	Do you want your ideas to be reflected in the research?	232 (47.9)	184 (38.0)	68 (14.1)	1 (0)
C14	Do you want to decide for yourself whether to continue participating in the study?	319 (66.1)	124 (25.7)	40 (8.3)	2 (0)

1) Participants who missed the school-age examination were excluded from these analyses (C-1/2: n = 93; C-3: n = 91).

Kappa coefficients for shared parent–child items were as follows: discussion of the study between parent and child (0.27, 95% CI: 0.21–0.33), changes in health or environmental awareness (0.26, 0.20–0.32), being accompanied to the examination (0.26, 0.19–0.33), impression of staff support (0.20, 0.09–0.31), impression of the newsletter (0.15, 0.09–0.20), discussion with others (0.11, 0.05–0.16), and willingness to talk about the study (0.02, −0.02–0.06) (Table 5).

**Table 5:** Parent–Child Agreement: Cohen’s kappa

**Question Number**		**Summary of Common Questionnaire Items**	**kappa Coefficient** **(95%CI)**
**Parent**	**Child**		
P3	C3	Enjoyed attending the school-age health examination together	0.26 (0.19–0.33)
P4	C2	Perceived the staff as courteous during the school-age examination	0.20 (0.09–0.31)
P5	C6	Looked forward to reading the JECS newsletter	0.15 (0.09–0.20)
P9	C9	Talked about the JECS study between parent and child	0.27 (0.21–0.33)
P12	C11	Talked about the JECS study with friends or other parents	0.11 (0.05–0.16)
P11	C12	Felt a desire to share or discuss experiences related to JECS	0.02 (−0.02–0.06)
P10	C10	Increased awareness of health or environmental issues through JECS	0.26 (0.20–0.32)

### Association between participation experience and retention intentions

Results of ordinal logistic regression are shown in Tables 6 (Parent) and 7 (Children).

**Table 6:** Adjusted odds ratios for parents’ retention intention by parental experience items

**Parental Variables**	**Odds Ratios (Adjusted)**	
	**Neutral vs Negative**	**Positive vs Negative**
P1 Questionnaire burden	1.91(1.03–3.55)	5.58(3.40–9.17)
P2 Scheduling burden	1.41(0.77–2.60)	3.76(2.21–6.41)
P3 Enjoying the visit with one’s child	0.61(0.08–4.63)	2.08(0.29–14.70)
P4 Staff courtesy	^1)^- (-–-)	^1)^- (-–-)
P5 News letter	1.65(0.88–3.07)	5.39(2.87–10.11)
P6 Media visibility	1.43(0.37–5.58)	8.64(1.55–48.04)
P7 Receiving study results	0.82(0.25–2.72)	2.51(0.92–6.87)
P8 Clarity of returned results	0.32(0.07–1.44)	1.26(0.29–5.47)
P9 Talking with one’s child	3.54(1.25–10.04)	3.74(2.22–6.30)
P10 One’s child’s increased interest	1.77(1.13–2.77)	4.30(2.50–7.42)
P11 Seeking peer connection	1.51(0.89–2.55)	3.38(2.13–5.39)
P12 Peer interaction	1.08(0.60–1.94)	1.59(0.76–3.29)
P13 Hoping parent networking	1.25(0.65–2.38)	2.77(1.37–5.63)
P17 Concern about potential disagreement	1.81(1.09–3.00)	5.75(3.51–9.42)
P18 Increased time demands	2.61(1.59–4.30)	5.80(3.50–9.63)
P19 Concerning about adolescence	0.93(0.56–1.57)	2.29(1.45–3.63)
Children Retention Intention	9.08(3.63–22.68)	35.47(15.06–83.55)

Note: Each adjusted odds ratio was estimated from a separate model including one parental experience item and four covariates (child’s gender, parent’s age, household income, and parental education). Children’s retention intention is shown as a reference variable for comparison. Analyses were based on complete cases for each model; sample size may vary by item due to missing responses.

1) There are no negative response on P4

**Table 7:** Adjusted odds ratios for children’s retention intention by children’s experience items

**Children Variables**	**Odds Ratios (Adjusted)**	
	**Neutral vs Negative**	**Positive vs Negative**
C1 Examination experience at venue	0.80(0.41–1.56)	2.45(1.42–4.22)
C2 Staff courtesy	17.37(0.42–720.97)	33.78(0.89–1278.88)
C3 Enjoying the visit with a parent	0.93(0.57–1.51)	1.31(0.86–1.99)
C4 Independent participation in the future	1.06(0.71–1.57)	3.49(2.00–6.09)
C5 Gift	2.36(0.87–6.39)	7.38(2.93–18.56)
C6 News letter	1.34(0.89–2.03)	4.22(2.45–7.27)
C7 JECS events	1.17(0.76–1.80)	5.73(3.50–9.38)
C8 Contributing to society	0.97(0.58–1.63)	3.71(2.25–6.12)
C9 Talking with parents	1.74(0.86–3.52)	3.16(2.14–4.68)
C10 Growing curiosity	1.44(0.93–2.23)	4.20(2.67–6.59)
C11 Peer interaction	2.10(1.27–3.48)	6.02(2.25–16.09)
C12 Seeking connection with peers	1.17(0.78–1.75)	6.72(3.39–13.29)
C13 Contributing ideas	1.17(0.69–1.99)	2.69(1.60–4.54)
C14 Self-determination in participation	0.91(0.46–1.81)	1.35(0.71–2.56)
Parent Retention Intention	12.20(3.33–44.64)	50.49(18.16–140.33)

Note: Each adjusted odds ratio was estimated from a separate model including one child experience item and four covariates (child’s gender, parent’s age, household income, and parental education). Parental retention intention is shown as a reference variable for comparison. Analyses were based on complete cases for each model; sample size may vary by item due to missing responses.

The proportional odds assumption was met in all models (Brant test; see Supplementary Script S1 for analysis script).

Among the parents respondents, a broadly graded pattern was observed across response categories: for all parent items except P9, the Negative–Positive contrast was larger than the Negative–Neutral contrast. Higher retention intention was associated with lower perceived burden related to questionnaire completion (P1; adjusted OR = 5.58, 95% CI: 3.40–9.17). Higher retention intention was also associated with positive experiences that may reflect perceived value of participation, including looking forward to the JECS newsletter (P5; 5.39, 2.87–10.11) and perceiving that participation had led their child to become more interested in health, society, or learning (P10; 4.30, 2.50–7.42). In addition, parents reporting fewer concerns about continued participation tended to report higher retention intention, including fewer concerns about potential disagreement with the child regarding continued participation (P17; 5.75, 3.51–9.42) and fewer concerns about difficulty continuing due to increased time demands in junior/high school (P18; 5.80, 3.50–9.63).

Among the children respondents, retention intention was most strongly associated with engagement and social factors, including anticipating the gift campaign (C5; adjusted OR = 7.38, 95% CI: 2.93–18.56), enjoying receiving the newsletter (C6; 4.22, 2.45–7.27), willingness to participate in events hosted by JECS (C7; 5.73, 3.50–9.38), reporting increased interest in health, society or environmental issues after participating in JECS (C10; 4.20, 2.67–6.59), having talked about the study with friends (C11; 6.02, 2.25–16.09), and interest in meeting peers from other schools participating in the study (C12; 6.72, 3.39–13.29). For several child items as well, the Negative–Positive contrast tended to exceed the Negative–Neutral contrast, consistent with a graded association across response categories.

Parent and child retention intentions were strongly associated. Parents tended to report higher retention intention when their child did (adjusted OR = 35.47, 95% CI: 15.06–83.55), and children likewise tended to report higher retention intention when their parent did (adjusted OR = 50.49, 95% CI: 18.16–140.33). The wide confidence intervals indicate that these estimates are imprecise and should be interpreted cautiously.

## Discussion

### Main findings

Retention intentions were reported by 92% of parents and 79% of children, parent–child responses showed broad overall alignment (quadratically weighted κ = 0.46), although disagreement was not negligible. Parental intention was associated mainly with lower burden and fewer concerns, whereas children’s intention was associated more strongly with emotional and social factors such as interest in events, gifts, and peer interaction. Although these patterns may appear intuitive when parent and child perspectives are considered separately, the present study adds to existing retention research by showing within the same parent–child pairs that motivations differ across generations even when overall orientations are broadly aligned. This suggests that retention in maturing birth cohorts should be understood not simply as a household-level preference, but as a relational process shaped by both shared family context and children’s emerging developmental perspectives.

### Retention intention and parent–child agreement

A majority of parents and many children reported retention intentions, and logistic regression suggested a positive association between the two. However, while concordance appeared high at a broad level, agreement was lower when assessed on the full five-point scale. This indicates that although parents and children generally point in the same direction, notable differences remain at a finer level of response.

### Participant experience and parent–child agreement

Parents most frequently reported positive experiences with accompanying their child to the school-age examination and with staff support. They also valued information-related aspects, such as interest in study results and clarity of explanations. Children likewise expressed satisfaction with staff support and being accompanied, but their interest was more often directed toward gift campaigns and events, with comparatively limited attention to newsletters or study information. These patterns suggest that parents may evaluate participation primarily in terms of practical support and informational value, whereas children may place greater weight on enjoyment and social opportunities.

Despite these generally positive impressions, concordance between parents and children on common questions was varied. (Table 5) Agreement was somewhat higher regarding whether they had spoken with each other about the study, but remained low for items such as impressions of the newsletter or discussions with others. In retention intention, parent–child responses showed broad overall alignment, with a quadratically weighted kappa of 0.46. For descriptive context, exact agreement on the original five-point scale was 53% (Table 2-2), whereas the 2–4 grade disagreement was 10% (50 pairs). These results are interpreted as overall moderate agreement but with non-negligible disagreement. This pattern may indicate that, as children mature, they increasingly form their own views about continued participation, even when their overall orientation remains similar to that of their parents.

Previous research indicates that discrepancies between parent and child reports are associated with adolescent adjustment and mental health [23, 24], raising concerns that similar gaps in perceptions of research participation may also have implications. In this context, the observed partial alignment suggests that although parents and children share broadly favorable views, they may differ in how they interpret specific aspects of participation. These differences emphasize the need to consider children as active participants whose perspectives do not necessarily mirror those of their parents.

### Association between retention intention and participation experience

The analysis indicated that parents’ and children’s retention intentions were associated with different sets of experiences. For parents, willingness to continue was greater when participation was less burdensome, such as completing questionnaires or arranging schedules. Retention intention was also associated with engagement-related experiences, including looking forward to the JECS newsletter. In addition, parents reporting that participation had led their child to become more interested in health, society, or learning tended to report higher retention intention. In contrast, concerns about continued participation—such as potential disagreement with the child and anticipated difficulty continuing as time demands increase during adolescence—have been recognized as potential challenges for retention in longitudinal studies [25, 26], and in our data were associated with lower parental retention intention. Children’s intentions were primary associated with experiential and social aspects. In our data, stronger child retention intention was associated with looking forward to the gift campaign, the newsletter, and JECS-hosted events, as well as talking with parents and developing greater interest in health or environmental issues. This pattern is consistent with prior work emphasizing children’s assent and families’ role in supporting children’s participation [25], as well as broader patient and public involvement frameworks that highlight engagement and reciprocity [27]. Parental and child intentions also appeared mutually associated, although the wide confidence intervals indicate imprecision and warrant cautious interpretation.

**Fig. 2:**
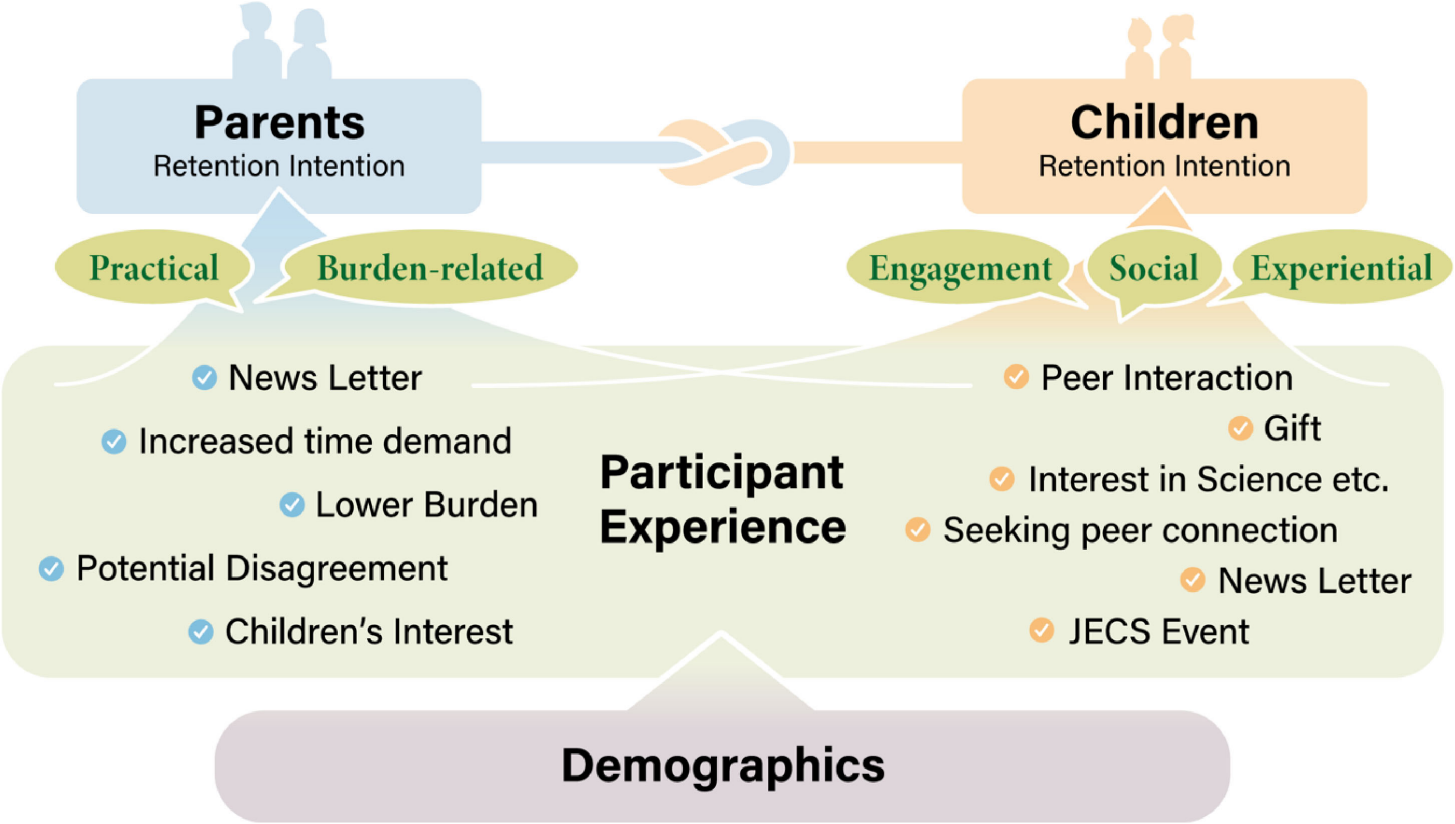
Conceptual summary of parent and child retention intentions and participation experiences Conceptual diagram illustrating how parent and child participation experiences may relate to their respective retention intentions, and how parent and child intentions may show broad alignment within pairs. The figure is intended as a schematic summary of hypothesized associations and does not imply causal or temporal relationships.

Overall, while both parents and children expressed considerable willingness to remain involved, their motivations diverged: parents emphasized practical burden and future concerns whereas children placed greater weight on engagement and social opportunities. These differences highlight the need for engagement strategies that address the distinct perspectives of each group. At the same time, the findings also suggest that these perspectives should not be understood in isolation. Taken together, these findings suggest that retention intention may be shaped through multiple interconnected pathways, including parent- and child-specific participation experiences, the association between parent and child intentions, and background factors such as sociodemographic characteristics. To aid interpretation, we added a conceptual framework figure (Fig. 2) summarizing hypothesized, non-causal relationships among these factors. Although the framework does not imply causality or temporal direction, it provides an overview of how these factors may interact and may help inform participant engagement strategies in other cohort settings.

### Sustained engagement design that includes children as well

In European cohort studies including those in the United Kingdom and Norway, increasing attention to participant engagement and involvement has supported efforts to integrate participant perspectives into study design and communication [10, 28]. In JECS, active PPI is still developing, though newsletters and local events reflect a participant-centered orientation. More broadly, scalable communication approaches—including digital methods [29] and tailored newsletters [30]—have been proposed to support engagement and retention, although their effectiveness may vary by population and context. This shift is also consistent with the 2024 Declaration of Helsinki [1], which refers to individuals involved in research as “participants” rather than “subjects,” underscoring respect for their agency and role in the research process. Growing attention to patient and public involvement (PPI) further suggests the importance of attending carefully to the experiences and perspectives of those involved in cohort research [28, 31]. Although this may appear fundamental, listening to participants’ voices can help build trust, improve the acceptability of study procedures and communication, and support sustained participation, thereby making cohort research not only more feasible but also more ethically grounded and methodologically robust. In this sense, the relevance of the present study is not limited to project-specific operational management, but extends to a broader issue in epidemiologic research: how attention to participants’ experiences may support continued participation and help maintain or improve overall study quality.

The contribution of the present study is therefore not primarily methodological, but participant-centered. To our knowledge, this perspective has been only rarely examined empirically within the main framework of large birth cohort studies. In this respect, the present study may be viewed as a modest step toward a more PPI-oriented perspective in long-term cohort research.

Within this broader perspective, the systematic inclusion of children’s perspectives remains limited. Participation in birth cohort studies begins with parental consent, but children’s views may diverge as they grow. In Japan, consent is legally required under age 16, and JECS decisions for 10–11-year-olds rely solely on parents. Evidence on how children perceive participation and how it affects family relationship remains scarce. While age-appropriate assent is increasingly emphasized internationally [25, 32], formal guidance in this area remains under development. Our findings can inform efforts to involve maturing children ethically, supporting assent processes and parent–child communication [20, 25, 32].

Overall, these results highlight the value of engagement measures while also underscoring the need to move beyond parent-centered approaches and recognize children as active contributors to cohort research.

### Limitations

This study has several limitations. First, its cross-sectional design precludes causal inference or directionality. While the primary aim was exploratory, reverse causation cannot be ruled out; for example, participants with stronger pre-existing commitment may retrospectively evaluate participation experiences more positively. Second, selection bias is possible due to the web-based format. Although internet penetration is high in Japan (>97% among adults aged 30–59), families with limited access or lower digital literacy may be underrepresented. Third, because the survey was restricted to the Kyoto Regional Center and the valid response rate was 44%, generalizability is limited. Direct respondent–non-respondent comparisons were not feasible because background information for non-respondents was not available. However, comparison with JECS-wide baseline distributions [33] suggests that our sample overrepresents families with higher education and household income. (Supplementary Table S1) Because this comparison was based on entry-period data collected in 2011–2014, it does not necessarily reflect representativeness at the time of the present survey. In addition, three-quarters of parents reported responding to regular questionnaires every year, indicating a highly engaged subgroup. Together, these features may overestimate overall retention intention and limit the applicability of findings to participants at highest risk of dropout. Importantly, those who did not respond to the survey may include participants with lower engagement and greater risk of attrition, meaning that the perspectives of those most vulnerable to dropout may not be adequately captured in the present study. Declining response rates remain a challenge in cohort research [33, 34]. Fourth, self-report measures are subject to parental reporting bias and social desirability bias, particularly regarding attitudes toward research participation. Moreover, although participants were instructed to answer independently, the parent and child completed the survey sequentially on the same device (parent → child → parent); thus, parental presence and differential privacy concerns for children may have influenced children’s responses. Future surveys may mitigate this by using separate devices, separate time windows, or settings that better ensure privacy. Finally, qualitative data from open-ended responses were not analyzed; incorporating these narratives in future work may enrich understanding of participant perspectives beyond quantitative findings.

## Conclusion

Both parents and children expressed willingness to continue participating in the cohort study, but their intentions were associated with different factors. Parents tended to emphasize cognitive and burden-related aspects, whereas children’s intentions seemed to be more strongly associated with emotional and social experiences. Our findings extend existing evidence on cohort retention and suggest new directions for designing engagement strategies that incorporate both parental and child perspectives.

## Abbreviations

JECS: Japan Environment and Children’s Study
PPI: Patient and Public Involvement
CROSS: Checklist for Reporting Of Survey Studies
STROBE: Strengthening the Reporting of Observational Studies in Epidemiology
OR: Odds Ratio
CI: Confidence Interval

## Supplementary information

**Additional file 1:** **Supplementary script S1:** R Code for the Brant Test Assessing the Proportional Odds Assumption in Ordinal Logistic Regression of Factors Associated with Parent’s Questionnaire Responses. **Supplementary Material 2:** R Code for the Brant Test Assessing the Proportional Odds Assumption in Ordinal Logistic Regression of Factors Associated with Children’s Questionnaire Responses.

**Additional file 2:** **Supplementary Table S1.** Comparison of Kyoto respondents with JECS-wide baseline distributions (mothers).

## Declarations

### Availability of data and material

The anonymized data supporting the findings of this study are available from the corresponding author upon reasonable request and with permission from the JECS.
