# Correction to: Parent and child experiences of research participation and retention intentions in a Japanese birth cohort: insights from the Japan Environment and Children’s Study (JECS)

**DOI:** 10.1265/ehpm.26-00239

**Published:** 2026-10-22

**Authors:** Tomoko Okabe, Yoshimitsu Takahashi, Mayumi Toyama, Yoshitaka Nishikawa, Kyoko Hirabayashi, Kumiko Kanatani, Fusako Niwa, Takeo Nakayama

**Affiliations:** 1 Department of Health Informatics, Kyoto University School of Public Health, Kyoto, Japan; 2 Department of Implementation Science in Public Health, Kyoto University School of Public Health, Kyoto, Japan; 3 Japan Environment and Children’s Study Kyoto Regional Center, Kyoto University Graduate School of Medicine, Kyoto, Japan

**Correction to: *Environ Health Prev Med* 31, 50 (2026)**

**https://doi.org/10.1265/ehpm.25-00355**

After the publication of this article [1], several corrections were identified in the number of eligible parents reported in the Abstract and in several entries in the Reference list. These corrections do not affect the results or conclusions of the article. Therefore, we correct them as follows.

**Abstract**

In the Results section of the Abstract, “Among 1,008 eligible parents of fifth-grade children” is corrected to “Among 1,108 eligible parents of fifth-grade children”.

**References**

The following references are corrected as follows:

2. Resneck J Jr. The Declaration of Helsinki Revision Process 2024. World Medical Association. Available from: https://www.wma.net/declaration-of-helsinki-revision-process-2024/ (Accessed 2026 Mar 13).
7. Kost RG, Lee LM, Yessis J, Coller BS, Henderson DK; Research Participant Perception Survey Focus Group Subcommittee. Assessing research participants’ perceptions of their clinical research experiences. Clin Transl Sci. 2011;4(6):403–413.
8. Yamamoto M, Fujita M, Mori C, Hata A. Survey of motivation to participate in a birth cohort. J Hum Genet. 2016;61:787–791.
15. von Elm E, Altman DG, Egger M, et al. The Strengthening the Reporting of Observational Studies in Epidemiology (STROBE) statement: guidelines for reporting observational studies. Epidemiology. 2007;18(6):800–804.
16. Kawamoto T, Nitta H, Murata K, et al. Rationale and study design of the Japan environment and children’s study (JECS). BMC Public Health. 2014;14:25.
20. Cayouette F, O’Hearn K, Gertsman S, et al. Operationalization of assent for research participation in pre-adolescent children: a scoping review. BMC Med Ethics. 2022;23:106.
27. Aas SN, Distefano MB, Pettersen I, et al. Patient and public involvement in health research in Norway: a survey among researchers and patient organisations. Res Involv Engagem. 2023;9:48.
30. Mohamedali M, Sandoval J, Thiruvarooran V, et al. Perceptions of Study Newsletters for Older Cancer Patients in Longitudinal Studies. J Cancer Educ. 2018;33(2):463–9.
31. Japan Agency for Medical Research and Development (AMED). Guidebook for PPI. Available from: https://www.amed.go.jp/content/000055212.pdf (Accessed 2026 Mar 13).
33. Michikawa T, Nitta H, Nakayama SF, et al. Baseline Profile of Participants in the Japan Environment and Children’s Study (JECS). J Epidemiol. 2018;28(2):99–104.
