# Supplementary material for: Parent and child experiences of research participation and retention intentions in a Japanese birth cohort: insights from the Japan Environment and Children’s Study (JECS)
Source: Environ Health Prev Med. 2026 Jul 25;31:50. doi: 10.1265/ehpm.25-00355 (PMC13413661; doi:10.1265/ehpm.25-00355)
Supplement: Supplementary file 1 — Additional file 1: Supplementary script S1: R Code for the Brant Test Assessing the Proportional Odds Assumption in Ordinal Logistic Regression of Factors Associated with Parent’s Questionnaire Responses. Supplementary Material 2: R Code for the Brant Test Assessing the Proportional Odds Assumption in Ordinal Logistic Regression of Factors Associated with Children’s Questionnaire Responses. [file ehpm-31-050-s001.docx]

Supplementary script S1: R Code for the Brant Test Assessing the Proportional Odds Assumption in Ordinal Logistic Regression of Factors Associated with Parent's Questionnaire Responses

# --- Load required packages ---

library(MASS) # For ordinal logistic regression (polr)

library(brant) # For Brant test to check proportional odds assumption

# --- Load data ---

data <- read.csv("~/Desktop/parent3.csv")

# --- Convert dependent variable to ordered factor ---

data$parent_intention <- factor(data$parent_intention, ordered = TRUE)

# --- List of explanatory variables (16 experience items) ---

experience_vars <- c("Item1", "Item2", "Item3", "Item4", "Item5", "Item6", "Item7", "Item8", "Item9", "Item10", "Item11", "Item12", "Item13", "item17", "item18", "item19", "item20")

# --- Initialize vector to store Omnibus p-values ---

omnibus_p_list <- c()

# --- Perform Brant test for each explanatory variable ---

for (var in experience_vars) {

formula_str <- paste("parent_intention ~", var,

"+ education_parent + income_parent + age_parent + sex_child")

tryCatch({

# Fit ordinal logistic regression model

model <- polr(as.formula(formula_str), data = data, Hess = TRUE)

# Perform Brant test

br <- brant(model)

# Capture printed output of Brant test

output <- capture.output(print(br))

# Extract line containing 'Omnibus' p-value

omni_line <- grep("Omnibus", output, value = TRUE)[1]

# Extract the last value (p-value) from the line

p_val <- as.numeric(tail(strsplit(omni_line, "\\s+")[[1]], 1))

# Store p-value

omnibus_p_list[var] <- p_val

# Display the result

cat(sprintf("\n--- %s : Omnibus p = %.3f ---\n", var, p_val))

}, error = function(e) {

cat(sprintf("\nError occurred with variable: %s\n", var))

omnibus_p_list[var] <- NA

})

}

# --- Display the complete list of Omnibus p-values ---

omnibus_p_list

# --- Session information for reproducibility ---

sessionInfo()

----------------------------------------------------

Test for X2 df probability

----------------------------------------------------

Omnibus 24.99 15 0.05

Item1 6.91 3 0.07

education_parent 2.87 3 0.41

income_parent 8.23 3 0.04

age_parent 0.9 3 0.82

sex_child 5.39 3 0.15

----------------------------------------------------

H0: Parallel Regression Assumption holds

--- Item1 Omnibus p = NA ---

----------------------------------------------------

Test for X2 df probability

----------------------------------------------------

Omnibus 20.95 15 0.14

Item2 2.03 3 0.57

education_parent 4.99 3 0.17

income_parent 7.38 3 0.06

age_parent 1.03 3 0.79

sex_child 4.45 3 0.22

----------------------------------------------------

H0: Parallel Regression Assumption holds

--- Item2 Omnibus p = NA ---

----------------------------------------------------

Test for X2 df probability

----------------------------------------------------

Omnibus 12.88 15 0.61

Item3 0.83 3 0.84

education_parent 3.81 3 0.28

income_parent 2.6 3 0.46

age_parent 3.3 3 0.35

sex_child 3.87 3 0.28

----------------------------------------------------

H0: Parallel Regression Assumption holds

--- Item3 Omnibus p = NA ---

----------------------------------------------------

Test for X2 df probability

----------------------------------------------------

Omnibus 11.9 15 0.69

Item4 0 3 1

education_parent 3.15 3 0.37

income_parent 2.53 3 0.47

age_parent 3.58 3 0.31

sex_child 3.78 3 0.29

----------------------------------------------------

H0: Parallel Regression Assumption holds

--- Item4 Omnibus p = NA ---

----------------------------------------------------

Test for X2 df probability

----------------------------------------------------

Omnibus 22.84 15 0.09

Item5 3.71 3 0.29

education_parent 4.16 3 0.24

income_parent 7.95 3 0.05

age_parent 0.87 3 0.83

sex_child 5.9 3 0.12

----------------------------------------------------

H0: Parallel Regression Assumption holds

--- Item5 Omnibus p = NA ---

----------------------------------------------------

Test for X2 df probability

----------------------------------------------------

Omnibus 5.46 15 0.99

Item6 0 3 1

education_parent 2.68 3 0.44

income_parent 1.93 3 0.59

age_parent 0.75 3 0.86

sex_child 0.81 3 0.85

----------------------------------------------------

H0: Parallel Regression Assumption holds

--- Item6 Omnibus p = NA ---

----------------------------------------------------

Test for X2 df probability

----------------------------------------------------

Omnibus 30.41 15 0.01

Item7 8.02 3 0.05

education_parent 3.61 3 0.31

income_parent 6.51 3 0.09

age_parent 0.53 3 0.91

sex_child 5.82 3 0.12

----------------------------------------------------

H0: Parallel Regression Assumption holds

--- Item7 Omnibus p = NA ---

----------------------------------------------------

Test for X2 df probability

----------------------------------------------------

Omnibus 24.1 15 0.06

Item8 2.9 3 0.41

education_parent 3.94 3 0.27

income_parent 8.58 3 0.04

age_parent 0.64 3 0.89

sex_child 5.14 3 0.16

----------------------------------------------------

H0: Parallel Regression Assumption holds

--- Item8 Omnibus p = NA ---

----------------------------------------------------

Test for X2 df probability

----------------------------------------------------

Omnibus 23.44 15 0.08

Item9 3.34 3 0.34

education_parent 3.87 3 0.28

income_parent 8.42 3 0.04

age_parent 0.71 3 0.87

sex_child 5.71 3 0.13

----------------------------------------------------

H0: Parallel Regression Assumption holds

--- Item9 Omnibus p = NA ---

----------------------------------------------------

Test for X2 df probability

----------------------------------------------------

Omnibus 18.56 15 0.23

Item10 0.04 3 1

education_parent 3.43 3 0.33

income_parent 8.04 3 0.05

age_parent 0.84 3 0.84

sex_child 6.11 3 0.11

----------------------------------------------------

H0: Parallel Regression Assumption holds

--- Item10 Omnibus p = NA ---

----------------------------------------------------

Test for X2 df probability

----------------------------------------------------

Omnibus 20.14 15 0.17

Item11 1.87 3 0.6

education_parent 3.56 3 0.31

income_parent 7.94 3 0.05

age_parent 0.9 3 0.83

sex_child 5.6 3 0.13

----------------------------------------------------

H0: Parallel Regression Assumption holds

--- Item11 Omnibus p = NA ---

----------------------------------------------------

Test for X2 df probability

----------------------------------------------------

Omnibus 20.56 15 0.15

Item12 1.57 3 0.67

education_parent 3.63 3 0.3

income_parent 8.22 3 0.04

age_parent 0.83 3 0.84

sex_child 5.94 3 0.11

----------------------------------------------------

H0: Parallel Regression Assumption holds

--- Item12 Omnibus p = NA ---

----------------------------------------------------

Test for X2 df probability

----------------------------------------------------

Omnibus 23.45 15 0.07

Item13 5.59 3 0.13

education_parent 4.04 3 0.26

income_parent 8.25 3 0.04

age_parent 0.92 3 0.82

sex_child 5.87 3 0.12

----------------------------------------------------

H0: Parallel Regression Assumption holds

--- Item13 Omnibus p = NA ---

----------------------------------------------------

Test for X2 df probability

----------------------------------------------------

Omnibus 20.75 15 0.14

item17 3.98 3 0.26

education_parent 5.04 3 0.17

income_parent 6.9 3 0.08

age_parent 0.96 3 0.81

sex_child 3.97 3 0.26

----------------------------------------------------

H0: Parallel Regression Assumption holds

--- item17 Omnibus p = NA ---

----------------------------------------------------

Test for X2 df probability

----------------------------------------------------

Omnibus 20.16 15 0.17

Item18 3.1 3 0.38

education_parent 4.96 3 0.17

income_parent 7.23 3 0.06

age_parent 0.72 3 0.87

sex_child 3.72 3 0.29

----------------------------------------------------

H0: Parallel Regression Assumption holds

--- Item18 Omnibus p = NA ---

----------------------------------------------------

Test for X2 df probability

----------------------------------------------------

Omnibus 17.85 15 0.27

Item19 1.12 3 0.77

education_parent 5.19 3 0.16

income_parent 5.38 3 0.15

age_parent 1.26 3 0.74

sex_child 4.19 3 0.24

----------------------------------------------------

H0: Parallel Regression Assumption holds

--- Item19 Omnibus p = NA ---

----------------------------------------------------

Test for X2 df probability

----------------------------------------------------

Omnibus 23.84 15 0.07

item20 6.39 3 0.09

education_parent 3.59 3 0.31

income_parent 7.05 3 0.07

age_parent 0.39 3 0.94

sex_child 5.13 3 0.16

----------------------------------------------------

H0: Parallel Regression Assumption holds

Supplementary Material 2: R Code for the Brant Test Assessing the Proportional Odds Assumption in Ordinal Logistic Regression of Factors Associated with Children's Questionnaire Responses

# --- Load required packages ---

library(MASS) # For ordinal logistic regression (polr)

library(brant) # For Brant test (proportional odds assumption)

# --- Load data ---

data <- read.csv("~/Desktop/child.csv")

# --- Convert dependent variable to ordered factor ---

data$child_intention <- factor(data$child_intention, ordered = TRUE)

# --- Ensure covariates have appropriate data types ---

data$age_parent <- as.numeric(data$age_parent)

# --- Variables to be tested (q1 to q15) ---

experience_vars <- paste0("q", 1:15)

# --- Initialize list to store Omnibus p-values from Brant test ---

omnibus_p_list <- list()

# --- Perform Brant test for each variable ---

for (var in experience_vars) {

# Define the formula for the ordinal logistic regression

formula <- as.formula(

paste("child_intention ~", var,

"+ sex_child + age_parent + income_parent + education_parent")

)

# Fit the ordinal logistic regression model

model <- polr(formula, data = data, Hess = TRUE)

# Perform Brant test

br <- brant(model)

# Capture the printed output of the Brant test

out <- capture.output(print(br))

# Extract the line containing 'Omnibus' p-value

omni_line <- grep("Omnibus", out, value = TRUE)[1]

# Split the line and extract the last element (p-value)

p_val <- as.numeric(tail(strsplit(omni_line, "\\s+")[[1]], 1))

# Store the p-value

omnibus_p_list[[var]] <- p_val

# Display result

cat(sprintf("%s → Omnibus p = %.3f\n", var, p_val))

}

# --- Display the complete list of Omnibus p-values ---

omnibus_p_list

# --- Session information for reproducibility ---

sessionInfo()

----------------------------------------------------

Test for X2 df probability

----------------------------------------------------

Omnibus 13.05 15 0.6

q1 2.59 3 0.46

sex_child 1.86 3 0.6

age_parent 7.13 3 0.07

income_parent 1.36 3 0.71

education_parent 0.93 3 0.82

----------------------------------------------------

H0: Parallel Regression Assumption holds

q1 → Omnibus p = 0.598

----------------------------------------------------

Test for X2 df probability

----------------------------------------------------

Omnibus 14.05 15 0.52

q2 6.22 3 0.1

sex_child 1.22 3 0.75

age_parent 7.23 3 0.06

income_parent 1.66 3 0.65

education_parent 1.01 3 0.8

----------------------------------------------------

H0: Parallel Regression Assumption holds

q2 → Omnibus p = 0.522

----------------------------------------------------

Test for X2 df probability

----------------------------------------------------

Omnibus 13.62 15 0.55

q3 3.1 3 0.38

sex_child 2.56 3 0.46

age_parent 6.96 3 0.07

income_parent 1.11 3 0.78

education_parent 0.93 3 0.82

----------------------------------------------------

H0: Parallel Regression Assumption holds

q3 → Omnibus p = 0.554

----------------------------------------------------

Test for X2 df probability

----------------------------------------------------

Omnibus 22.99 15 0.08

q4 15.78 3 0

sex_child 0.93 3 0.82

age_parent 4.3 3 0.23

income_parent 0.95 3 0.81

education_parent 0.81 3 0.85

----------------------------------------------------

H0: Parallel Regression Assumption holds

q4 → Omnibus p = 0.084

----------------------------------------------------

Test for X2 df probability

----------------------------------------------------

Omnibus 12.71 15 0.62

q5 4.81 3 0.19

sex_child 1.1 3 0.78

age_parent 4.67 3 0.2

income_parent 0.82 3 0.84

education_parent 0.89 3 0.83

----------------------------------------------------

H0: Parallel Regression Assumption holds

q5 → Omnibus p = 0.625

----------------------------------------------------

Test for X2 df probability

----------------------------------------------------

Omnibus 13.57 15 0.56

q6 6.03 3 0.11

sex_child 0.94 3 0.82

age_parent 4.27 3 0.23

income_parent 0.77 3 0.86

education_parent 0.81 3 0.85

----------------------------------------------------

H0: Parallel Regression Assumption holds

q6 → Omnibus p = 0.558

----------------------------------------------------

Test for X2 df probability

----------------------------------------------------

Omnibus 24.14 15 0.06

q7 16.53 3 0

sex_child 0.56 3 0.91

age_parent 4.23 3 0.24

income_parent 0.81 3 0.85

education_parent 1 3 0.8

----------------------------------------------------

H0: Parallel Regression Assumption holds

q7 → Omnibus p = 0.063

----------------------------------------------------

Test for X2 df probability

----------------------------------------------------

Omnibus 9.1 15 0.87

q8 1.56 3 0.67

sex_child 0.98 3 0.81

age_parent 4.63 3 0.2

income_parent 0.38 3 0.95

education_parent 0.9 3 0.83

----------------------------------------------------

H0: Parallel Regression Assumption holds

q8 → Omnibus p = 0.872

----------------------------------------------------

Test for X2 df probability

----------------------------------------------------

Omnibus 23.88 15 0.07

q9 15.92 3 0

sex_child 0.79 3 0.85

age_parent 4.32 3 0.23

income_parent 0.99 3 0.8

education_parent 1.19 3 0.75

----------------------------------------------------

H0: Parallel Regression Assumption holds

q9 → Omnibus p = 0.067

----------------------------------------------------

Test for X2 df probability

----------------------------------------------------

Omnibus 10.29 15 0.8

q10 2.68 3 0.44

sex_child 0.77 3 0.86

age_parent 4.31 3 0.23

income_parent 0.79 3 0.85

education_parent 0.73 3 0.87

----------------------------------------------------

H0: Parallel Regression Assumption holds

q10 → Omnibus p = 0.801

----------------------------------------------------

Test for X2 df probability

----------------------------------------------------

Omnibus 11.61 15 0.71

q11 4.47 3 0.21

sex_child 1.38 3 0.71

age_parent 4.34 3 0.23

income_parent 0.78 3 0.85

education_parent 0.81 3 0.85

----------------------------------------------------

H0: Parallel Regression Assumption holds

q11 → Omnibus p = 0.708

----------------------------------------------------

Test for X2 df probability

----------------------------------------------------

Omnibus 9.05 15 0.87

q12 1.69 3 0.64

sex_child 0.92 3 0.82

age_parent 4.29 3 0.23

income_parent 0.7 3 0.87

education_parent 0.87 3 0.83

----------------------------------------------------

H0: Parallel Regression Assumption holds

q12 → Omnibus p = 0.875

----------------------------------------------------

Test for X2 df probability

----------------------------------------------------

Omnibus 11.75 15 0.7

q13 3.43 3 0.33

sex_child 0.81 3 0.85

age_parent 4.79 3 0.19

income_parent 0.71 3 0.87

education_parent 0.91 3 0.82

----------------------------------------------------

H0: Parallel Regression Assumption holds

q13 → Omnibus p = 0.698

----------------------------------------------------

Test for X2 df probability

----------------------------------------------------

Omnibus 16.3 15 0.36

q14 8.84 3 0.03

sex_child 1.01 3 0.8

age_parent 4.43 3 0.22

income_parent 0.55 3 0.91

education_parent 0.73 3 0.87

----------------------------------------------------

H0: Parallel Regression Assumption holds

q14 → Omnibus p = 0.363

----------------------------------------------------

Test for X2 df probability

----------------------------------------------------

Omnibus 10.28 15 0.8

q15 3.7 3 0.3

sex_child 0.69 3 0.87

age_parent 4.25 3 0.24

income_parent 1.11 3 0.77

education_parent 1.48 3 0.69

----------------------------------------------------

H0: Parallel Regression Assumption holds

q15 → Omnibus p = 0.802
