# Supplementary material for: Parent and child experiences of research participation and retention intentions in a Japanese birth cohort: insights from the Japan Environment and Children’s Study (JECS)
Source: Environ Health Prev Med. 2026 Jul 25;31:50. doi: 10.1265/ehpm.25-00355 (PMC13413661; doi:10.1265/ehpm.25-00355)
Supplement: Supplementary file 2 — Additional file 2: Supplementary Table S1. Comparison of Kyoto respondents with JECS-wide baseline distributions (mothers). [file ehpm-31-050-s002.docx]

Supplementary Table S1. Comparison of Kyoto respondents with JECS-wide baseline distributions (mothers)

| **Education (years) ‡** | **Kyoto (n/N*)** | **Kyoto (%)** | **JECS-wide mothers (%)†** |
| --- | --- | --- | --- |
| <10 | 8 / 477 | 1.7 | 4.8 |
| 10–12 | 47 / 477 | 9.9 | 31.5 |
| 13–16 | 387 / 477 | 81.1 | 62.2 |
| ≥17 | 35 / 477 | 7.3 | 1.5 |
| **Household income (million JPY/year)** | **Kyoto (n/N*)** | **Kyoto (%)** | **JECS-wide mothers (%)†** |
| <2 | 7 / 408 | 1.7 | 5.7 |
| 2 to <4 | 29 / 408 | 7.1 | 34.6 |
| 4 to <6 | 80 / 408 | 19.6 | 33.1 |
| 6 to <8 | 93 / 408 | 22.8 | 15.9 |
| 8 to <10 | 98 / 408 | 24.0 | 6.5 |
| ≥10 | 101 / 408 | 24.8 | 4.3 |

**†** JECS-wide baseline distributions are from Michikawa et al. (2018), Table 2 (mothers, 2011–2014).

**‡** To align Kyoto respondents’ educational background with JECS “years of education” categories, Kyoto education was approximated as follows: junior high school → <10 years; high school → 10–12 years; technical college/junior college/vocational school/university → 13–16 years; graduate school → ≥17 years.

* Percentages for Kyoto respondents were calculated excluding “prefer not to answer” and missing responses (education: N=477, excluding 7 “prefer not to answer” and 1 missing; income: N=408, excluding 67 “prefer not to answer” and 10 missing).
